# Patient and relative understanding of Martha’s Rule: identifying barriers to patient-activated escalation in a mature system

**DOI:** 10.3389/frhs.2026.1812956

**Published:** 2026-07-08

**Authors:** Alexander Jeffrey, Eirian Edwards, Manohar Joishy, Christian Subbe

**Affiliations:** 1 North Wales Medical School, Bangor University, Bangor, United Kingdom; 2 School of Medicine, Cardiff University, Cardiff, United Kingdom; 3 Ysbyty Gwynedd, Betsi Cadwaladr University Health Board, Bangor, United Kingdom

**Keywords:** critical care outreach, Martha’s Rule, patient safety, public awareness, rapid response system

## Abstract

Martha’s Rule allows patients and families to escalate concerns about safety to Critical Care Outreach teams in real time. This service evaluation aimed to investigate understanding of patients and their families in two key settings; paediatric and adult secondary care. We undertook an opportunistic survey of 168 patients and relatives recruited at a single District General Hospital in North Wales, where patient and family activated escalation had been in place for more than two years and was widely advertised throughout the hospital and through communication in regional and national media. 93% of the interviewees were unaware of the service. Further barriers identified were confusion around terminology and the need for such a service, and fear of perceived “whistleblowing”. We explored the differences found in understanding between patients and relatives on adult or paediatric wards. Our findings suggest the need for a review of strategy of the present national information campaign.

## 1 Introduction

Rapid response systems have been in place since the early 2000s, despite this, the management of critically ill patients remains a challenge faced globally. Welch et al. (1) estimate that failure to manage patients with clear signs of clinical deterioration accounts for 620 avoidable deaths each month in England alone, with 7% of reported deaths in acute hospitals relating to failure to recognise or respond to deterioration (2).

Patients and their families often have the most personal understanding of their condition and are frequently the first to notice deterioration; however, these concerns can go unheard. Martha's Rule is an NHS England and Wales policy initiative that follows the preventable death of a teenage girl, Martha Mills. This service, or its implementation as “Call for Concern,” advocates for patient and family involved care by allowing for direct escalation to Critical Care Outreach teams, who aim to provide care for ward-based patients at risk of critical deterioration. Existing models already in place globally include Ryan's Rule, Call for Help, and REACH across Australia (3).

Difficulty remembering the escalation procedure when deteriorating limits activation by patients and families (4), and data on patient awareness of these new services is sparse. Therefore, this service evaluation aimed to determine the level of understanding of Martha's Rule in a local setting in adult patients and families of paediatric patients.

## 2 Methods

### 2.1 Sampling

The Ysbyty Gwynedd is a District General Hospital in rural North Wales with 500 beds across all major specialties. It was the first hospital in Wales to introduce a patient activated rapid response service across all adult wards in April 2023, following an intensive communication campaign involving leaflets, posters and postings on national journals (5) and television (6). All participants interviewed from this hospital were informed explicitly by the same interviewer, not affiliated with the Critical Care Outreach team, that they had the right to decline completion of the interview.

250 participants were approached across 14 different wards from 02/06/2025 to 13/06/2025. Selection was pragmatic based on availability of patients and relatives, with the exception of those who were unable to read the written information provided and those who declined participation, giving an overall response rate of 67.2%. We aimed to interview a similar number of patients and relatives between wards; however, other possible confounding variables including age and gender were not removed and were instead analysed separately.

### 2.2 Procedure

Participants were interviewed using a standardised questionnaire (available in Supplementary Material) designed for the purpose of this study, piloted with a smaller sample of patients in the same hospital. The questionnaire consisted of five binary questions and four further optional open questions. Questions explored five key elements of understanding: prior knowledge, when the escalation service is indicated, how to use the service, regular escalation procedure and understanding of the terminology “clinical deterioration”. These elements were measured as a surrogate marker for the successful application of Martha's Rule in an acute setting while also allowing us to pinpoint individual areas to target improved understanding. During the interview, participants were offered to read an information leaflet designed by the local Critical Care Outreach team to ensure an equal level of access to information before measuring understanding. Participants were also asked about their personal barriers to understanding and suggestions for improvement.

Participants' gender, ward, and age group were recorded. All participants provided answers to the closed questions; 35% of participants provided additional extended answers to the optional questions.

### 2.3 Analysis

Data was analysed using the Statistical Package for Social Sciences (SPSS, version 29; IBM Corp). Data was tested for significance using simple descriptive analysis with a high-level qualitative approach.

### 2.4 Ethics

This service evaluation was registered and approved with the Ysbyty Gwynedd audit department. Ethics approval was not required. All participants were informed by the interviewer that they had the right to decline completion of the interview. Children were interviewed only in the presence of their parents. Data was collected anonymously in accordance with the General Data Protection Regulations (7).

## 3 Results

### 3.1 Population

168 participants aged between 13 and 91 were interviewed. Of these, 95 were adult patients, 22 were relatives of adult patients and 51 were relatives of paediatric patients, including 1 child. 55% (*n* = 93) of interviewees were women. 40 interviews were conducted on surgical wards, 55 on medical wards, 51 in paediatrics and 22 in other or mixed wards. 47.6% of interviewees were >70 years old, and a small minority of the participants were relatives of patients receiving outpatient care (5.4%).

### 3.2 Quantitative data

Of the 168 participants interviewed, 6.5% (*n* = 11) stated existing knowledge of patient activated escalation, with healthcare workers accounting for 6 of these. Participants' understanding of when to activate the service, measured after reading the written information, was 83.3% (*n* = 140), with 81.5% (*n* = 137) then understanding who to contact when feeling unwell. Further to this, 79% (*n* = 132) showed understanding of how to contact the Critical Care Outreach team. 24.4% of participants (*n* = 41) had no appreciation of the meaning of the term “clinical deterioration”. Table 1 outlines these data against different age groups (See Table 1).

**Table 1:** Heatmap of responses of understanding between age groups.

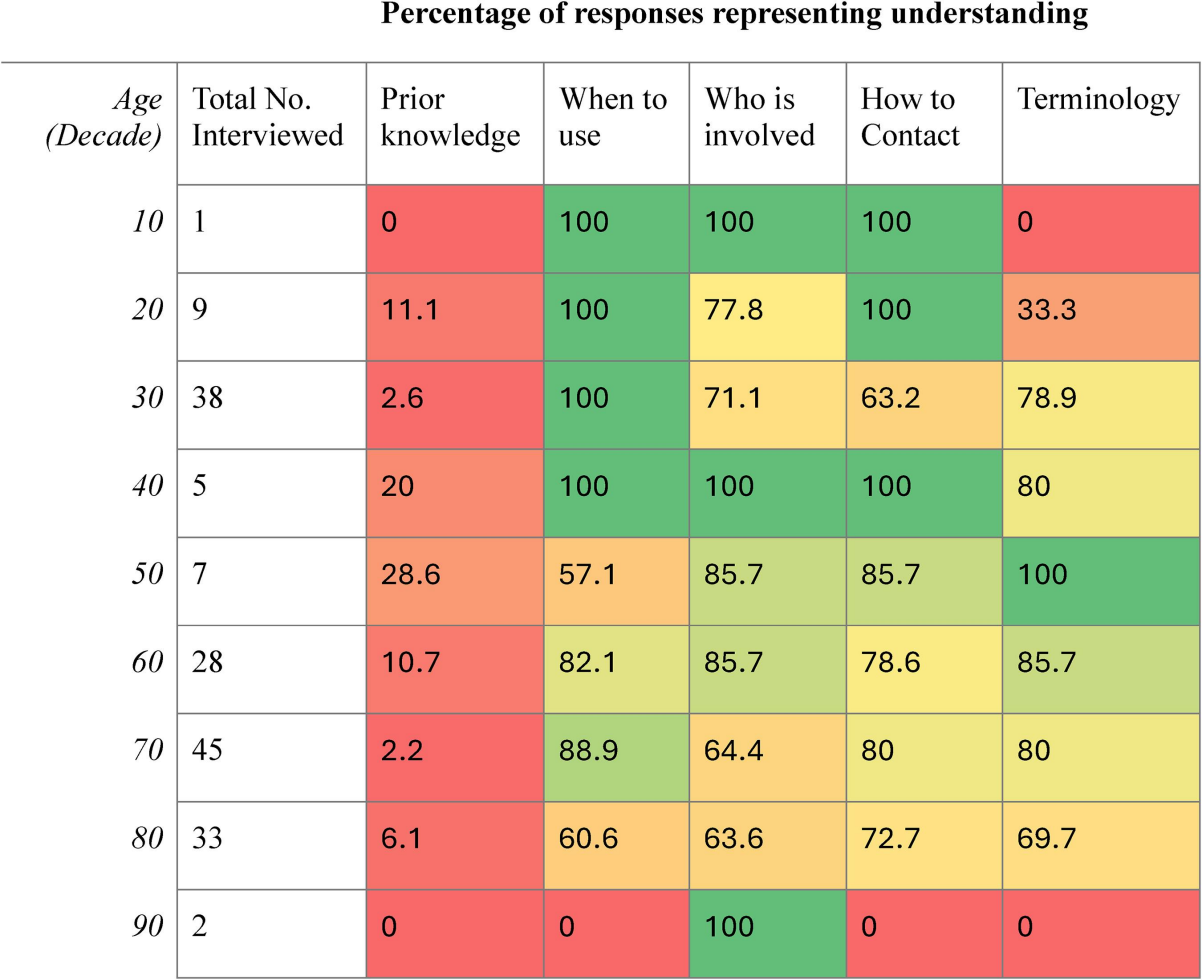						

The percentage of responses that represent an understanding of each element of the interview across different ages, grouped into respective decades. Higher average understanding across groups is shown in green with lower understanding shown in red.

**Figure 1:**
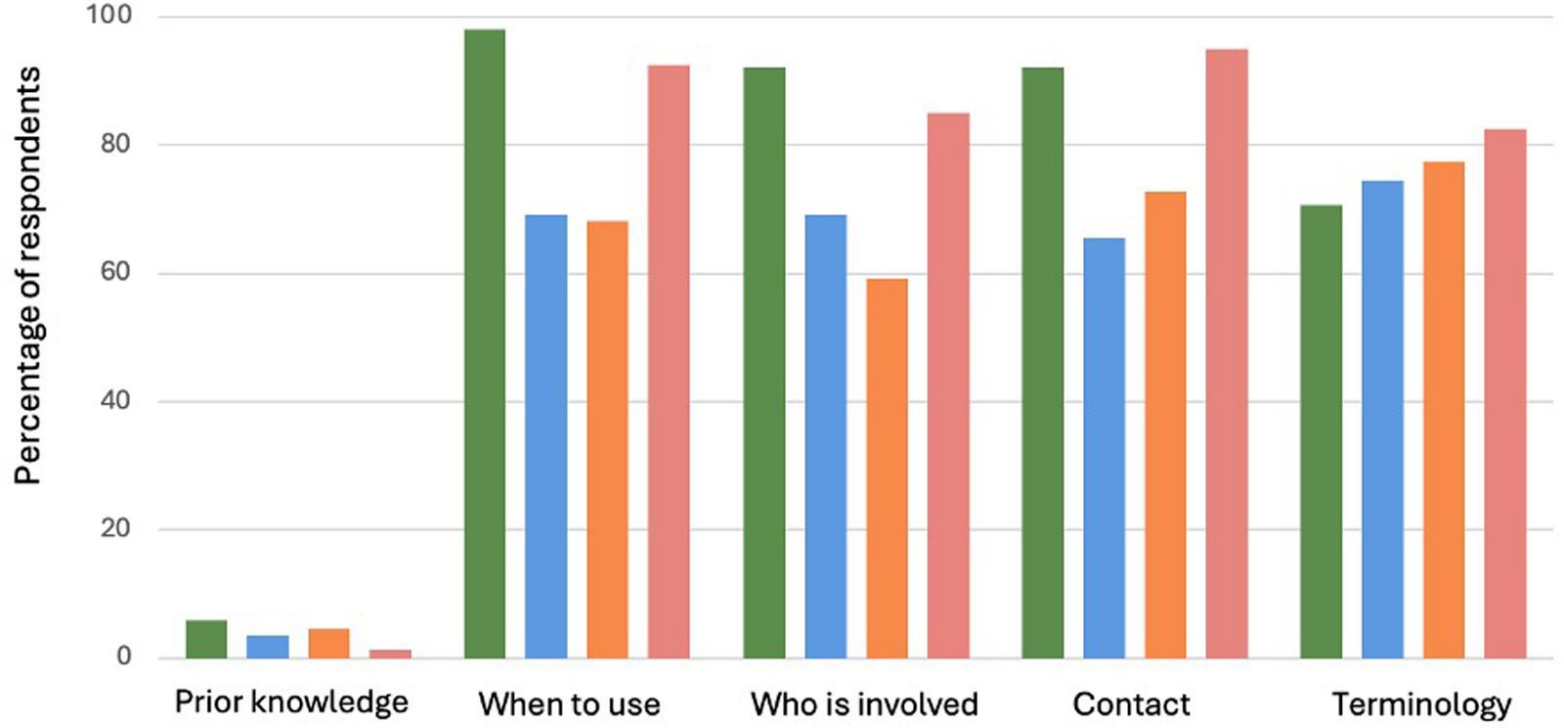
Measures of understanding across different wards. Understanding is shown as a percentage for each element of the interview in four designated ward types: paediatric (green), adult medical (blue), mixed (orange) and surgical (red).

Figure 1 summarises differences in measured understanding between paediatric and adult wards (See Figure 1). We were also interested in quantifying any differences in interpretation between genders that may suggest a tendency to use gendered communication when providing information to patients and relatives. We found only one significant difference in how to contact the team between ward groups, while prior knowledge, understanding who to contact and the term “clinical deterioration” between both ward and gender showed no significant differences.

### 3.3 Replies to optional open questions

59 patients provided more detailed replies to questions: When asked about possible barriers in understanding patient-activated escalation, many individuals cited an inability to read the written information available as a key limitation. One respondent (30s, male) who had dyslexia expressed difficulty in reading the leaflet for the interview. Several others were unable to read themselves due to pre-existing vision impairment; after having the information read aloud to these participants, their level of comprehension was similar to those who were able to read the information themselves.

Possible limitations in patient-activated escalation also became apparent, largely surrounding fear of negative consequences from staff who are initially caring for the patient and how they might receive the news of the service being used. “I would be hesitant to use this because I don’t want the staff to treat my child any worse if they see the team come to review him” (mother, 30s). “My main concern is that it feels like whistleblowing to complain about the care, and that would put me off using it” (mother, 30s).

Substantial confusion about the need for such a service was also evident. One participant (70s, male) explained, “If I was given information on this, I wouldn't take any notice of it, because I'm in hospital and I don't expect to deteriorate around all of these [healthcare] staff”. This was further compounded by confusion around the chosen name of the local service, “Call for Concern,” “The name pangs a little bit of ‘Neighbourhood Watch,’ not something I would expect in a hospital” (80s, female). “The name makes me think that the service is for if you're concerned about an elderly relative living at home and you want someone to go and look after them” (80s, female).

Responses from participants reaffirmed the findings from quantitative data analysis of an urgent need to increase public awareness; seven interviewees conveyed that they would have already used this service if they had known about it sooner. Additionally, a further two expressed a desire to be told the information in the leaflet on admission to hospital.

## 4 Discussion

This service evaluation aimed to explore understanding of patient-activated escalation in patients and relatives in paediatric and adult inpatient care. The results indicate a severe lack of public awareness of this patient-initiated service, even in a hospital with over two years of experience with the service, following an intense communication campaign. A general lack of significance in difference also showed that understanding was limited across the groups sampled within this hospital. Overall, our data suggests that the most obvious shortcoming in the understanding of patient-activated escalation lies with the limited awareness of the public, as well as some concerns about complex language.

It is important to consider that a sizeable number of patients and relatives still lacked understanding of some or all elements of successful activation of Martha's Rule, these areas of lower overall understanding highlight possible inequalities that have not been considered, such as use of terminology or ambiguous wording that particular subsets of the general population may find more confusing than others. From this, we are able to conclude that the existing information made available is not beyond reproach, and improvements must continue to be made at a regional level concurrently with ongoing public awareness campaigns.

Given the weight placed on being able to read the written information, as well as the number of patients who were unable to participate in the evaluation due to an inability to read, this presents a clear opportunity moving forward to produce auditory- or graphical-based information alternatives to expand the number of patients and relatives who are informed enough to understand and therefore activate Martha's Rule. It is also pertinent to note that a significant proportion of patients were confused and delirious and thus unable to contribute. We estimate that approximately one-third of patients and relatives invited to participate were a part of this group, where possible, relatives of these patients were included in the evaluation, however it is critical to ensure that these patients are treated equally and given access to perhaps simpler variations of information as it becomes available.

Conducting this service evaluation in a single hospital restricts the conclusions we can draw from these results; repeating this evaluation across multiple sites would therefore be important in validating the generalisability of our results. However, limitations to patient-activated initiatives are frequently cited by existing literature. Welch et al. (1) identify limitations in the sole use of vital signs as a predictive measure, as well as the fallible nature of recording and responding to these alone. Furthermore, Albutt et al. (2) cite poor communication from healthcare staff as the commonest issue causing concern for patients and relatives. While understanding is not the only limitation in activation of Martha's Rule, with other more complex reasons including contextual, professional and cultural factors holding significant weight (8), only by appreciating the entire multifaceted sphere of barriers are we then able to account for and correct these.

A desire to receive information during admission is also not a finding isolated to this study; Ntumba et al. (4) support this finding with interviews of their own patient cohort. Featherstone et al. (9) further reinforce our results showing confusion to be a key barrier in understanding; they found that resistance to bedside care was commonplace for people with dementia, further emphasising not only the need to inform patients earlier, but also the need for active relative involvement.

The most obvious barrier to the future impact of Martha's Rule we found is the widespread lack of prior awareness. Therefore, future clinical practice would benefit from improved public awareness of this service using multichannel communication and increased media presence, at a local level, this will include continued efforts to provide information through publicly available methods, such as news articles and forums, as well as social media campaigns to reach an even greater number of the general public, especially in younger age groups. These efforts should also be combined with widened accessibility to information, to remove any inequalities in understanding and eradicate confusion and fear around the service that exists in the local population.

## 5 Conclusion

Our findings raise concern that the impact of Martha's Rule will be limited by a lack of public awareness. This is an inherent limitation in timely activation of this service and should be swiftly remedied to capitalise on patients' and relatives' keen cognisance of self or loved ones' conditions.

Our results also indicate a need for more widely accessible information. This may require the development of alternative auditory-based information sources, together with information that avoids the use of complex terminology.

For Martha's Rule to have wide-reaching success, healthcare workers must become advocates for the service, informing patients and relatives not only of its existence but also providing clear instructions on how and when to activate, regardless of age, gender, or perceived levels of understanding. In the interim before the development of alternative forms of information, this may be done through word of mouth or by signposting to currently available information, while still recognising that this is not infallible.

## Data Availability

The raw data supporting the conclusions of this article will be made available by the authors, without undue reservation.

## References

[1] Welch J Murkin J Edwards E Inada-Kim M Subbe C. Giving patients, families and staff a reliable voice in acute care: expert guidance for implementation of Martha’s Rule in UK hospitals. Future Healthcare J. (2025) 12(1):2514–6645. 10.1016/j.fhj.2025.100223 PMC11889965 40059870

[2] Albutt A O’Hara J Conner M Fletcher S Lawton R. Is there a role for patients and their relatives in escalating clinical deterioration in hospital? A systematic review. Health Expect. (2017) 20(5):818–25. 10.1111/hex.12496 27785868 PMC5600219

[3] Bucknall T Quinney R Booth L McKinney A Subbe C Odell M. When patients (and families) raise the alarm: patient and family activated rapid response as a safety strategy for hospitals. Future Healthcare J. (2021) 8(3):e609–12. 10.7861/fhj.2021-0134 PMC8651329 34888450

[4] Ntumba M Edwards E Haegdorens F Subbe C. Patient activated rapid response – the ‘999’ for patients admitted to hospital. J Patient Saf Risk Manag. (2023) 28(4):156–62. 10.1177/25160435231199338

[5] Dyer C. Martha’s Rule: what could the proposed changes mean for doctors? Br Med J. (2023) 382:2067. 10.1136/bmj.p2067 37684028

[6] ITV News. ‘It’s OK to ask’ – Calls for Welsh ‘Martha’s Rule’ backed by grieving mum (2024). Available online at: https://www.itv.com/news/wales/2024-03-27/grieving-mum-backs-calls-to-seek-second-opinions-in-hospital (Accessed September 3, 2025).

[7] General Data Protection Regulation. (2016). Available online at: https://gdpr-info.eu/ (Accessed September 3, 2025).

[8] Braaten J. Hospital system barriers to rapid response team activation: a cognitive work analysis. Am J Nurs. (2015) 115(2):22–32. 10.1097/01.NAJ.0000460672.74447.4a 25588088

[9] Featherstone K Northcott A Bridges J. Routines of resistance: an ethnography of the care of people living with dementia in acute hospital wards and its consequences. Int J Nurs Stud. (2019) 96:53–60. 10.1016/j.ijnurstu.2018.12.009 30679033

