# Supplementary material for: Patient and relative understanding of Martha’s Rule: identifying barriers to patient-activated escalation in a mature system
Source: Front Health Serv. 2026 Jul 8;6:1812956. doi: 10.3389/frhs.2026.1812956 (PMC13388555; doi:10.3389/frhs.2026.1812956)
Supplement: Supplementary file 1 [file Table1.docx]

Supplementary Material

**Appendix: Questionnaire**

A.1 Patient survey

Ward –

Age –

Relation to patient –

Q1. Have you heard of Call-4-Concern / Martha’s Rule?

Q2. Do you understand when to use the Call-4-Concern?

Q3. Do you know who you would speak to if you are feeling unwell?

Q4. Do you understand how to contact the team?

Q5. Do you understand what is meant by the term ‘clinical deterioration’?

A.2 Extended Questions

Q1. What does the name ‘Call-4-Concern’ make you think it is for?

Q2. After reading the leaflet, is there anything that you are still unsure about?

Q3. What do you think could limit someone’s understanding and them using this service?

Q4. What changes would you like to see / Do you think would improve understanding of Call-4-Concern?
